# GhSNAP33, a t-SNARE Protein From *Gossypium hirsutum*, Mediates Resistance to *Verticillium dahliae* Infection and Tolerance to Drought Stress

**DOI:** 10.3389/fpls.2018.00896

**Published:** 2018-07-03

**Authors:** Ping Wang, Yun Sun, Yakun Pei, Xiancai Li, Xueyan Zhang, Fuguang Li, Yuxia Hou

**Affiliations:** ^1^College of Science, China Agricultural University, Beijing, China; ^2^State Key Laboratory of Cotton Biology, Institute of Cotton Research of The Chinese Academy of Agricultural Sciences, Anyang, China

**Keywords:** cotton (*Gossypium hirsutum*), GhSNAP33, *Verticillium dahliae*, drought stress, transgenic *Arabidopsis*, resistance

## Abstract

Soluble *N*-ethylmaleimide-sensitive fusion protein attachment protein receptor (SNARE) proteins mediate membrane fusion and deliver cargo to specific cellular locations through vesicle trafficking. Synaptosome-associated protein of 25 kDa (SNAP25) is a target membrane SNARE that drives exocytosis by fusing plasma and vesicular membranes. In this study, we isolated *GhSNAP33*, a gene from cotton (*Gossypium hirsutum*), encoding a SNAP25-type protein containing glutamine (Q)b- and Qc-SNARE motifs connected by a linker. *GhSNAP33* expression was induced by H_2_O_2_, salicylic acid, abscisic acid, and polyethylene glycol 6000 treatment and *Verticillium dahliae* inoculation. Ectopic expression of *GhSNAP33* enhanced the tolerance of yeast cells to oxidative and osmotic stresses. Virus-induced gene silencing of *GhSNAP33* induced spontaneous cell death and reactive oxygen species accumulation in true leaves at a later stage of cotton development. *GhSNAP33*-deficient cotton was susceptible to *V. dahliae* infection, which resulted in severe wilt on leaves, an elevated disease index, enhanced vascular browning and thylose accumulation. Conversely, *Arabidopsis* plants overexpressing *GhSNAP33* showed significant resistance to *V. dahliae*, with reduced disease index and fungal biomass and elevated expression of *PR1* and *PR5*. Leaves from *GhSNAP33*-transgenic plants showed increased callose deposition and reduced mycelia growth. Moreover, *GhSNAP33* overexpression enhanced drought tolerance in *Arabidopsis*, accompanied with reduced water loss rate and enhanced expression of *DERB2A* and *RD29A* during dehydration. Thus, GhSNAP33 positively mediates plant defense against stress conditions and *V. dahliae* infection, rendering it a candidate for the generation of stress-resistant engineered cotton.

## Introduction

Eukaryotic cells including those of plants contain membrane-enclosed organelles that communicate through vesicle trafficking and exchange (Steegmaier et al., 1998; Jahn and Scheller, 2006). This process delivers cargo to specific locations in the cell via a membrane fusion event such as exo- or endocytosis, and is critical for cell growth and division and for maintaining the spatial organization of biochemical reactions (McNew et al., 2000; Bonifacino and Glick, 2004). Soluble *N*-ethylmaleimide-sensitive fusion protein attachment protein receptor (SNARE) proteins mediate membrane fusion at each step of the secretory pathway (Bock et al., 2001; Jahn and Scheller, 2006). Selective membrane fusion is achieved through interactions between SNAREs located on vesicles and on target membranes (v- and t-SNAREs, respectively) (McNew et al., 2000). A typical SNARE complex contains glutamine (Q)a-, Qb-, and Qc- and arginine (R)-SNARE motifs that forms a tetrameric bundle of coiled helices (Ernst and Brunger, 2003; Bassham and Blatt, 2008).

Synaptosome-associated protein of 25 kDa (SNAP25)-type proteins are the best known isoform of the t-SNARE subfamily and mediate the fusion of vesicles with the plasma membrane during exocytosis (Fukuda et al., 2000). These proteins contain Qb and Qc SNARE domains connected via an anti-parallel linker (Steegmaier et al., 1998; Wang et al., 2008). SNAP25-type proteins play important roles in various organisms. The yeast homologs Sec9 and Spo20 function in secretion and sporulation, respectively (Fukuda et al., 2000; Strop et al., 2008). Of the four mammalian SNAPs (SNAP23, SNAP25, SNAP29, and SNAP47) (Holt et al., 2006), SNAP25 is a component of the synaptic SNARE complex that mediates synaptic vesicle fusion and exocytosis (Nagy et al., 2008). SNAP23 regulates phagosome formation and maturation in macrophages, with its loss delaying maturation of and reducing uptake by phagosomes (Sakurai et al., 2012). Loss of SNAP29 impairs endocytic recycling and cell motility, resulting in cerebral dysgenesis, neuropathy, ichthyosis, and keratoderma syndrome (Rapaport et al., 2010). SNAP47 is widely distributed on intracellular membranes of neurons and can replace SNAP-25 in SNARE complex formation (Holt et al., 2006). PtSNAP, the *Paramecium* homolog of metazoan SNAP-25, shows several divergent features including resistance to cleavage by botulinum neurotoxins (Schilde et al., 2008). SNAP25 depletion in the Gulf Coast tick impaired feeding and engorgement and prevented oviposition (Browning and Karim, 2013). AtSNAP33 is the first characterized SNAP25-type protein in plants and regulates cytokinesis in *Arabidopsis* via interaction with KNOLLE (Heese et al., 2001), and is also involved in the response to pathogens and mechanical stimulation (Wick et al., 2003). The SNARE family proteins penetration (PEN)1, SNAP33, and vesicle-associated membrane protein (VAMP)721/722 constitute an immune secretory pathway in plant defense that modulates immune responses through focal secretion (Collins et al., 2003; Kwon et al., 2008b; Yun et al., 2016; Yun and Kwon, 2017). *OsSNAP32* is involved in the response to polyethylene glycol (PEG) 6000 and low temperature stress and may enhance rice resistance against blast fungus (Bao et al., 2008b; Luo et al., 2016). HvSNAP34, a SNAP25-type protein in barley, associates with syntaxin (ROR2) and contributes to powdery mildew resistance (Collins et al., 2003). *Cynanchum komarovii* *CkSNAP33* enhances *Arabidopsis* disease resistance to *Verticillium dahliae* (Wang et al., 2017), and *Glycine soja* *GsSNAP33* increases tolerance to salt and drought stress in transgenic *Arabidopsis* (Nisa et al., 2017).

Cotton is a widely grown agricultural and industrial crop of considerable economic value in the textile industry (Sunilkumar et al., 2006; Gao et al., 2011). Significant effort has been expended to increase the sustainable yield and quality of cotton by improving plant cultivars and cultivation practices (Hill et al., 1999; Wang et al., 2011a; Zhang et al., 2012; Zhao et al., 2012). The recent availability of genome sequences not only provides genetic resources to study complex genome evolution, but also allows the exploitation of genetic resources for improvement of cotton agricultural performance under conditions of biotic and abiotic stress (Wang et al., 2012; Li et al., 2014, 2015; Zhang T. et al., 2015). Additionally, the development of *Agrobacterium*-mediated virus-induced gene silencing (VIGS) has facilitated the investigation of cotton gene function and has contributed to the dissection of the stress response in cotton (Gao et al., 2011; Gao X. et al., 2013; Cox et al., 2017).

Verticillium wilt caused by the soil-borne fungal pathogen *V. dahliae* Kleb is among the most prevalent and lethal diseases in cotton (Daayf et al., 1995; Fradin and Thomma, 2006; Klosterman et al., 2009). *V. dahliae* colonizes the plant through young, uninjured roots or puncture wounds to the xylem and causes browning of the vasculature, leaf discoloration, wilting, and defoliation (Garas et al., 1986). Verticillium wilt is difficult to control due to the viability and persistence of *V. dahliae* microsclerotia in soil (Fradin and Thomma, 2006) and shortage of resistance cotton germplasms (Yang et al., 2008). It has been reported that cotton phenylpropanoid pathway, terpenoid pathway, salicylic acid, reactive oxygen species and jasmonic acid signaling pathways are important contributors to *V. dahliae* response (Ashraf et al., 2018). In addition, many other cotton genes were shown to be required for resistance to *V. dahliae*, such as *GhHb1* (Qu et al., 2005), *GhNDR1*, *GhMKK2* (Gao et al., 2011), *GhPGIP* (Liu et al., 2017), *GhPMEI3* (Liu et al., 2018), and receptor like protein or kinase genes *GbaVd1* and *GbaVd2* (Chen et al., 2017), *GhBAK1* (Gao X. et al., 2013), *Gh-LYK1* and *Gh-LYK2* (Gu et al., 2017). Among different abiotic stresses, drought stress is a major factor affecting cotton production. Many studies have examined the genetic basis of the drought stress response (Li et al., 2017; Ma et al., 2017) and ways to enhance cotton drought tolerance (Guo et al., 2017; Mishra et al., 2017; Zahoor et al., 2017). Meanwhile, Several drought-related genes including transcription factors *GhWRKY59* (Li et al., 2017), *GhDERB2* (Li et al., 2017), *GhERF38* (Ma et al., 2017), *GhNAC79* (Guo et al., 2017) and *GhABF* (Kerr et al., 2017) and *GhAnn1* (Zhang F.et al., 2015) have been reported in cotton. However, none of vesicle trafficking related genes has been characterized in cotton so far and their contributions to cotton stress and disease defense responses remains elusive.

To this end, in the present study we isolated *GhSNAP33*—the first SNARE family gene identified in cotton (*Gossypium hirsutum*)—encoding a SNAP25-type protein. *GhSNAP33* expression in response to various types of stress was characterized in cotton and via ectopic expression in yeast (*Saccharomyces cerevisiae*). The VIGS assay was used to evaluate the function of *GhSNAP33* in cotton development and defense against *V. dahliae* infection. We also evaluated the role of *GhSNAP33* in disease resistance and drought tolerance in transgenic *Arabidopsis* plants. These findings advance our understanding of the function plant SNAP25-type protein in both biotic and abiotic stress and may facilitate the development cotton with improved adaptability to different environment.

## Materials and Methods

### Plant, Yeast, and *V. dahliae* Cultivation

*Gossypium hirsutum* L. cultivar Zhongzhiming 2 (Verticillium wilt-resistant upland cotton) seeds were provided by the Cotton Research Institute, Chinese Academy of Agricultural Sciences and germinated in pots filled with a mixture of soil and vermiculite (2:1, w/w) in a growth chamber under 16-h light (25°C)/8-h dark (22°C) conditions. *Arabidopsis* seeds (Columbia ecotype) were used in this study. After vernalization for 3 days at 4°C, the seeds were geminated in pots containing a mixture of soil and vermiculite (1:1, w/w) in a chamber under 16-h light (22°C)/8-h dark (20°C) conditions. *S. cerevisiae* strain INVSC1 (genotype MATα-ahis3Δ1 leu2 trp1-289, ura3-52) was used as the yeast host cell. The highly aggressive defoliating isolate Vd991 of *V. dahliae* was cultured on potato dextrose agar at 25°C for 7 days, and then inoculated in Czapek liquid medium. After 7 days, the suspension was harvested by filtration through four layers of cheesecloth and adjusted to a concentration of 10^6^ conidia per mL for inoculation.

### Gene Cloning and Sequence Analyses

Total RNA was isolated from cotton seedlings using a kit (Promega, Madison, WI, United States) according to the manufacturer’s instructions. The PolyATract mRNA Isolation System (Promega) was used to generate polyadenylated mRNA. The cDNA library was prepared as previously described (Wang et al., 2011b; Liu et al., 2017) and propagated on 140 mm plates to obtain about 10^6^ clones. The conserved region of SNAP33 (Heese et al., 2001; Wang et al., 2017) was used as a probe to screen for positive clones (Liu et al., 2016; Wang et al., 2017).

The theoretical isoelectric point (pI) and molecular mass were calculated with ProtParam^1^. A transmembrane hidden Markov model (TMHMM) analysis of the transmembrane domain was performed using the TMHMM online tool^2^. Multiple amino acid sequence alignment was performed with Clustal Omega^3^, and the multiple alignment file was shaded with BoxShade^4^. A motif analysis was performed using the National Center for Biotechnology Information (NCBI) conserved domain search program^5^. A phylogenetic tree was constructed with the neighbor-joining method using MEGA 6, with bootstrap values from 1000 replicates indicated at the nodes.

### Analysis of *GhSNAP33* Expression

Two-week-old cotton seedlings were gently uprooted from soil and cleaned with water for treatment. The seedlings were placed in 10% (w/v) PEG, 100 μM abscisic acid (ABA), 1 mM salicylic acid (SA), or 10 mM H_2_O_2_ solution or inoculated with *V. dahliae*. For pathogen treatment, seedling roots were inoculated with a *V. dahliae* conidial suspension for 3 min and the seedlings were transplanted into pots with fresh soil. Control samples were treated with sterile water. Three plants were combined for RNA isolation at each time point of each treatment condition. The experiment was repeated three times.

Real-time PCR was performed to detect transcript levels of *GhSNAP33* using SYBR Premix Ex Taq (Tli RNaseH Plus) (Takara Bio, Dalian, China) on an ABI 7500 thermocycler (Applied Biosystems, Foster City, CA, United States) under the following conditions: 95°C for 30 s, followed by 40 cycles of 95°C for 5 s and 60°C for 34 s. A pair of primers (qGhSNAP33-F and qGhSNAP33-R) was designed to amplify a fragment of the *GhSNAP33* gene, and *GhUBQ7* (DQ116441) was used as an internal reference gene that was amplified with primers qUBQ-F/qUBQ-R [26]. The relative transcript level of *GhSNAP33* was calculated with the comparative 2^-ΔΔCt^ method.

### The Generation of Transgenic Plants

The full-length *GhSNAP33* was cloned using primers ZW33-F/ZW33-R with *Xba*I/*Sal*I restriction sites at the 5′ and 3′ ends, respectively. The resultant PCR fragment was inserted into a modified pCAMBIA 1300 vector harboring a hygromycin phosphotransferase (*hptII*) gene and the green fluorescent protein (GFP) gene under the control of the super promoter (Wang et al., 2011b). The vector was introduced into *Arabidopsis* Columbia ecotype via *Agrobacterium* *tumefaciens* (strain GV3101)-mediated transformation. Transgenic *Arabidopsis* seeds were screened on MS plates containing 25 μg/mL hygromycin B and the genomic DNA was extracted for verification by PCR using the vector-specific primers 1300-F/1300-R. Semi-quantitative RT-PCR was performed to confirm *GhSNAP33* expression. The primers are listed in Supplementary Table S1.

### Yeast Transformation and Stress Tolerance Assays

The *GhSNAP33* gene was amplified by PCR with primers pYES-GhSNAP33-F/pYES-GhSNAP33-R and introduced into the pYES2.0 vector between the *EcoR*I/*Xho*I restriction sites. *S. cerevisiae* strain INVSC1 was transformed with the pYES2.0 and pYES-GhSNAP33 plasmids using the lithium acetate method (Lee et al., 1999; Bao et al., 2008a; An et al., 2011). Total RNA was isolated from the cells and *GhSNAP33* expression was evaluated by semi-quantitative RT-PCR. The growth rate of transformed yeast cells was monitored under various conditions as previously described (An et al., 2011). Briefly, the cells were cultured overnight and the medium was changed to SC-ura plus 2% galactose for 24 h. The optical density at 600 nm was adjusted at 0.4 with medium containing 100 mM H_2_O_2_ or 0.75 M mannitol, followed by culturing for 24 h. 3 μL serial diluted cells were spotted onto SC-ura agar medium. The assay was repeated at least three times with similar results.

### Agrobacterium-Mediated VIGS

Total RNA was isolated from cotton seedlings using EASYspin Fast Plant RNA kit (Biomed, Beijing, China) according the manufacturer’s instructions. First-strand cDNA was synthesized using the FastQuant RT kit (Tiangen, Beijing, China). Fragments of *GhCLA1* and *GhSNAP33* were amplified from cotton cDNA and inserted into the pTRV2 vector by ligation-independent cloning (Dong et al., 2007). The ligation product was transformed into *Escherichia coli* DH5α cells. Plasmids from positive transformants were tested by PCR analysis and sequencing.

The pTRV1 (pYL192), pTRV2-*GhCLA1*, and pTRV2-*GhSNAP33* plasmids were transformed into *A. tumefaciens* strain GV310 by heat shock. *Agrobacterium* clones positive for pTRV1, pTRV2-*GhCLA1*, or pTRV2-*GhSNAP33* were inoculated in Luria–Bertani (LB) broth supplemented with 50 mg/L kanamycin and 50 mg/L rifampicin and cultured overnight at 28°C. The culture was expanded in fresh LB medium containing 50 mg/L kanamycin, 50 mg/L rifamycin, 10 mM 2-(*N*-morpholino) ethanesulfonic acid, and 20 μM acetosyringone. Bacterial collection, pretreatment, and infiltration were carried out as previously described (Gao et al., 2011).

The efficiency of *GhSNAP33* and *GhCLA1* silencing was evaluated by RT-PCR. Two weeks after *Agrobacterium* infiltration—i.e., when *GhCLA1*-silenced plants showed signs of albinism—the second true leaf of each plant was harvested for RNA isolation. The vGh33F/R and vGhCLA1 F/R primers were used for RT-PCR; *GhUBQ7* from cotton was amplified as an internal control with primers qUBQ-F and qUBQ-R. The experiment included three biological repeats.

### Histochemical Analysis of H_2_O_2_ Production, Cell Death and Callose Deposition

3,3′-Diaminobenzidine (DAB) was used to detect H_2_O_2_ in cotton leaf tissue (Xiao et al., 2003; Gao X. et al., 2013). Detached leaves were incubated in 1 mg/mL DAB-HCl (pH 3.8) (Sigma-Aldrich, St. Louis, MO, United States) in the dark for 8 h. The leaves were fixed and cleared by boiling in alcoholic lactophenol (95% ethanol: lactophenol, 2:1 v/v) for 20 min. Trypan blue staining was performed as previously described to detect cell death in leaves (Gao X. et al., 2013; Liu et al., 2017). Briefly, detached leaves were stained by boiling in lactophenol-Trypan Blue (10 mL lactic acid, 10 mL glycerol, 10 g phenol, and 40 mg trypan blue dissolved in 10 mL distilled water) and then immersed in choral hydrate solution (250% w/v) to remove chlorophyll. The stained leaves were observed and images of cotton leaves were acquired with a Nikon digital camera. Callose deposition on *Arabidopsis* was stained by aniline blue as previous (Meyer et al., 2009). For *Arabidopsis* leaves, cell death and mycelia growth were observed and imaged under an optical microscope (Nikon ECLIPSE Ti, Tokyo, Japan) and callose deposition were imaged on fluorescence microscopy (Nikon C1). At least eight leaves from each VIGS cotton or *Arabidopsis* plants were evaluated, and the experiment was repeated at least three times.

### Inoculation of *V. dahliae* and Disease Detection

Three weeks after *Agrobacterium*-mediated VIGS, true leaves of *GhCLA1*-silenced cotton plants showed clear signs of albinism. The plants were inoculated with *V. dahliae* as previously reported (Gao W. et al., 2013; Zhang et al., 2017). Briefly, the seedlings were uprooted from the soil and their roots were immersed in the *V. dahliae* spore suspension (1 × 10^6^ conidia/mL) for 3 min. The seedlings were then replanted in soil and cultured in a moist growth chamber. The disease index was calculated based on three repeats, each comprising at least 15 plants. Two weeks after inoculation, slices of fresh stems approximately 1 cm below the cotyledon were collected and examined under an optical microscope (ECLIPSE Ti; Nikon).

The disease phenotype of transgenic *Arabidopsis* plants was examined. Three-week-old transgenic and wild-type (WT) *Arabidopsis* plants were inoculated with *V. dahliae* spores as previously described (Gao X. et al., 2013). The disease index and fungal biomass were calculated (Wang et al., 2017). The primers used for the quantification of *V*. *dahliae* were qVd-F/qVd-R. Real-time PCR was performed as previous to determine the transcription level of the genes pathogenesis-related protein 1 (*PR1*) and pathogenesis-related protein 5 (*PR5*) in *V. dahliae* infected *Arabidopsis* at 6 dpi. The specific primers for *PR1* and *PR5* were qPR1-F/qPR1-R and qPR5-F/qPR5-R, respectively. *AtEF1-*α (NM_100666.3) was used as the endogenous control and was detected using the primer pair AtEF1α-F/AtEF1α-R (the sequences of the primers mentioned here are listed in Supplementary Table S1).

The fungal filtrate assay was performed as reported (Thatcher et al., 2009; Chen et al., 2016), with minor modification. Leaves detached from 3-week-old *Arabidopsis* plants were placed adaxial side up on moist filter paper in Petri dishes and 5 μL of *V. dahliae* spores suspension (10^7^ conidia/mL) were applied to each leaf. The dishes were sealed with Parafilm and incubated at 25°C in a moist chamber. Trypan Blue staining was performed to visualize *V. dahliae* mycelia and assess cell death at 6 dpi. Callose deposition was detected at 24 h post inoculation.

### Transmission Electron Microscopy (TEM)

For TEM observation of thylose accumulation, the *V. dahliae*-infected control and *GhSNAP33*-silenced cotton roots were washed with distilled water and sliced vertically into less than 1 mm thick. These slices were fixed immediately in 2.5% glutaraldehyde, washed with 0.1 M PBS (pH 7.4) buffer and post-fixed with 1% osmium tetroxide. After dehydration with a graded acetone series (30, 50, 80, 90, and 100%), the slices were embedded in Spurr’s resin mixture. Ultrathin serial sections were cut from resin blocks, followed by uranyl acetate and lead citrate staining. The samples were sectioned for TEM analysis with JEM-1230 electron microscope (JEOL, Ltd., Japan).

### Plant Drought Treatment

*Arabidopsis* drought treatment was carried out as previously described (Shi et al., 2013; Li et al., 2017). Briefly, water was withheld from 3-week-old *Arabidopsis* plants for 20 days, followed by watering for 2 days; the survival rate was then recorded. The experiment was repeated three times with 18 plants each.

For the water loss test, detached aerial parts of 3-week-old *Arabidopsis* seedlings were placed on filter paper on a bench in the growth chamber. The decrease in fresh weight was monitored at indicated times (Liu et al., 2013; Li et al., 2017). The DREB2A and RD29A transcript level was testes by real-time PCR in *Arabidopsis* plants at 1 h after dehydration. The assay was repeated with at least three different batches, with each line containing 10 plants as one set.

## Results

### Characterization of GhSNAP33

**FIGURE 1:**
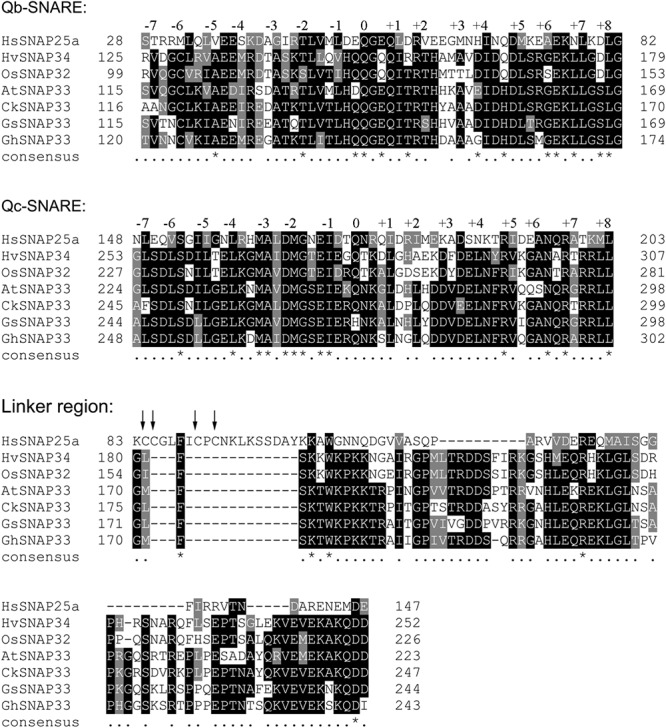
Alignment of conserved domains of GhSNAP33 and other SNAP25-type t-SNARE proteins. HsSNAP25a (AAH10647.1) from *Homo sapiens*, OsSNAP32 (AAW82752.1) from *Oryza sativa*, HvSNAP34 (AAP79417.1) from *Hordeum vulgare*, AtSNAP33 (Q9S7P9.1) from *Arabidopsis thaliana*, *CkSNAP33* (KR011961) from *Cynanchum komarovii* and GsSNAP33 from *Glycine soja*. Conserved and similar residues are shaded in black and gray, respectively. The well conserved heptad repeat layers –7 to +8 are indicated. The four cysteine residues involved in palmitoylation of SNAP25 are indicated using arrow. The asterisks (^∗^) and dots (.) under the sequence are indicated the completely conserved and highly conserved amino acids, respectively. Multiple amino acid sequence analyses were performed using Clustal Omega and the multiple alignment file was shaded using the BoxShade program.

A synaptosome-associated protein was isolated from *G. hirsutum* by colony *in situ* hybridization and named GhSNAP33 (GenBank accession number: KR011955). The DNA sequence of GhSNAP33 was 2281 bp with five exons and four introns (Supplementary Figure S1A) and a 915 bp open reading frame encoding a 305 amino acid protein (Supplementary Figure S1B) with a theoretical pI of 6.27 and molecular weight of 33.75 kDa. GhSNAP33 had no transmembrane domain or signal peptide. An analysis of the functional domains of GhSNAP33 with other known SNAP25-type proteins revealed that a conserved Qb-SNARE was located at amino acids 120–174 and Qc-SNARE domain was located at amino acids 248–302 of the C terminus and a relatively conserved linker region among all the SNAP25 protein from plants (**Figure 1**). Q145 and Q273 of GhSNAP33 contributed to the formation of the zero ionic layer and heptad repeat layers engaged in a hydrophobic interaction with Qa- and R-SNARE (**Figure 1**). Different from the HsSNAP25a, neither of these plant SNAP25 contains palmitoylation sites (**Figure 1**).

**FIGURE 2:**
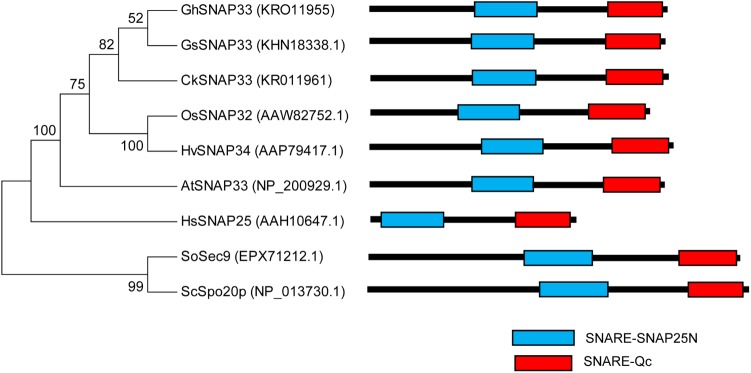
Phylogenetic analysis and domain structures of GhSNAP33 and known SNAP25-type proteins. A phylogenetic tree was constructed using the neighbor-joining method in MEGA v.6.06 and bootstrap values from 1,000 replicates are indicated at the nodes. The conserved SNARE domain was predicted via NCBI CDD search.

A phylogenic analysis of several functional SNAP25-type proteins showed that GhSNAP33 formed a cluster with GsSNAP33 and had a close genetic relationship to CkSNAP33 (**Figure 2**) indicating that they may have similar function. A conserved domain analysis confirmed that all the SNAP25-type homolog contains tandem Qb- and Qc-SNARE motifs comprising 64–65 and 59 amino acids, respectively. The linker region is different on the account of species, plant SNAP25-type protein linker region is comprising of 70–71 amino acids; linker of Sosec9 and Scspo20p from yeast is 89 and 81 amino acids; however, the linker region in HsSNAp25 is shorter with 60 amino acids (**Figure 2**). The N-terminal region of HsSNAP25 is much shorter and that of the two proteins from yeast is than those of plant SNAP25 homologs (**Figure 2**).

### *GhSNAP33* Transcription Is Activated by Various Stresses

**FIGURE 3:**
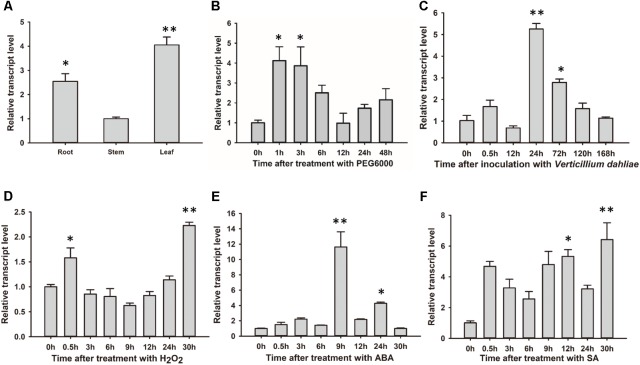
*GhSNAP33* mediates crosstalk between various stress responses in cotton. **(A)** Tissue-specific expression of GhSNAP33 in cotton. **(B–F)** GhSNAP33 expression in the presence of 10% (w/v) PEG 6000 **(B)**, following *V. dahliae* inoculation **(C)**, and upon treatment with 10 mM H_2_O_2_ **(D)**, 100 μM ABA **(E)**, or 1 mM salicylic acid **(F)**. Results were pooled from three independent biological replicates. Data are shown as mean ± standard error of three independent experiments. ^∗^*P* < 0.05, ^∗∗^*P* < 0.01 vs. control.

*GhSNAP33* was most highly expressed in cotton leaf, with higher levels observed in the root than in the stem (**Figure 3A**). The change in *GhSNAP33* expression in response to various stresses was investigated. *GhSNAP33* transcription was induced with PEG6000, ABA, SA, or H_2_O_2_ treatment and plants were infected with *V. dahliae* at different time points. Treatment with 10% (w/v) PEG6000 markedly increased gene expression at 1 and 3 h (**Figure 3B**). *GhSNAP33* expression was upregulated at 24 and 72 h after *V. dahliae* infection (**Figure 3C**). *GhSNAP33* transcription was slightly increased in the presence of 10 mM H_2_O_2_ at 0.5 h and then declined to a normal level before increasing at 30 h (**Figure 3D**). *GhSNAP33* level was highly increased at 9 and 24 h of treatment with 100 μM ABA (**Figure 3E**), whereas 1 mM SA caused *GhSNAP33* expression to increase at 12 h, with a maximum level observed at 30 h (**Figure 3F**).

### *GhSNAP33* Transformed Yeast Exhibits Stress Tolerance

**FIGURE 4:**
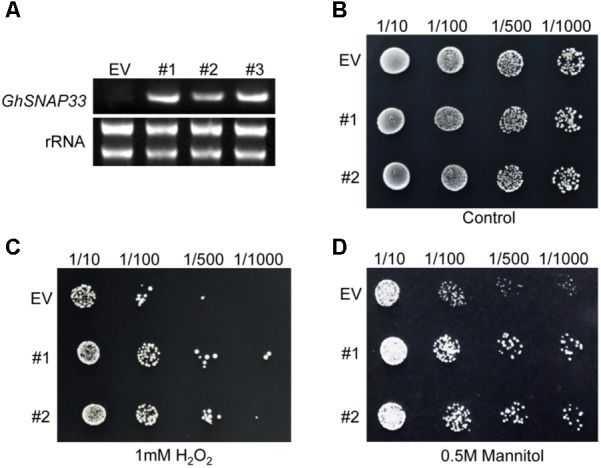
Stress tolerance in yeast cells overexpressing *GhSNAP33*. **(A)** RT-PCR analysis of GhSNAP33 expression in yeast cells. EV, yeast cells transformed with the pYES2.0 empty vector; #1, #2, and #3, yeast cell lines transformed with the pYES2.0-GhSNAP33 vector. **(B)** Growth serially diluted yeast cells (1/10, 1/100, 1/500, and 1/1000 dilutions). **(C)** Growth of yeast cells (1/10, 1/100, 1/500, and 1/1000 dilutions) after 1 mM H_2_O_2_ treatment. **(D)** Growth of yeast cells (1/10, 1/100, 1/500, and 1/1000 dilutions) after 0.5 M mannitol treatment. These assays were repeated three times with similar results.

*GhSNAP33* expression was confirmed in transformed yeast cells by semi-quantitative RT-PCR; cells transformed with the empty vector pYES2.0 served as a control (**Figure 4A**). Serially diluted cultures showed no differences in colony density between *GhSNAP33*-transformed and control cells (**Figure 4B**). Cells expressing *GhSNAP33* showed an increase in colony density following treatment with 1 mM H_2_O_2_ as compared to the control (**Figure 4C**). In the presence of 0.5 M mannitol, *GhSNAP33* transformants had a higher colony density (**Figure 4D**). These results demonstrate that ectopic *GhSNAP33* expression enhances yeast tolerance to H_2_O_2_ and mannitol, suggesting that GhSNAP33 enables yeast cells to adapt to oxidative and osmotic conditions.

### GhSNAP33 Is Involved in Cotton Development

**FIGURE 5:**
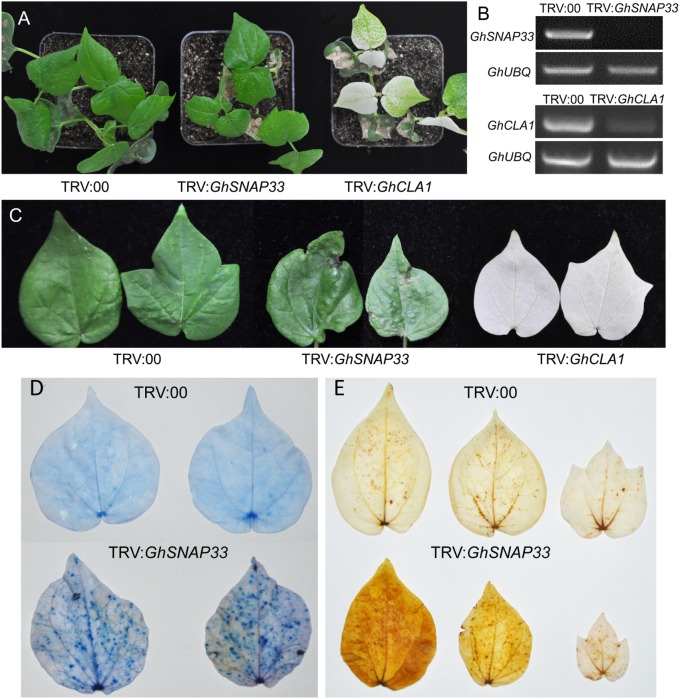
Cell death and ROS production induced by *GhSNAP33* silencing. **(A)** Growth of cotton plants 2 weeks after *Agrobacterium*-mediated VIGS. **(B)** Evaluation of *GhSNAP33* knockdown efficiency by semi-quantitative RT-PCR. GhUBQ was served as a control. **(C)** Cotton leaves from VIGS-deficient plants 4 weeks after *Agrobacterium* infiltration. **(D)** Cell death in true leaves induced by *GhSNAP33* silencing, as determined by trypan blue staining. Leaves were detached and stained 4 weeks after VIGS. **(E)** GhSNAP33 silencing induces ROS production, as detected by DAB staining. Leaves were detached and stained 4 weeks after VIGS.

To clarify the role of GhSNAP33 in cotton development, we generated VIGS-*GhSNAP33* cotton seedlings. Expression of *CLOROPLASTOS ALTERADOS* (*GhCLA1*), a gene involved in chloroplast development (Gao et al., 2011), was used as a visual marker to monitor VIGS efficiency. The semi-quantitative RT-PCR confirmed that transcription of *GhSNAP33* and *GhCLA1* was reduced at 2 weeks after VIGS (**Figure 5B**).

At 2 weeks post infiltration, *GhSNAP33*-slienced plants were smaller than controls (**Figure 5A**) and senior true leaves became abnormally curved and had black spots at 4 weeks (**Figure 5C**). Trypan blue staining revealed an increase in the number of blue dots in second true leaves of *GhSNAP33*-inoculated cotton, while nearly no dots were observed on the leaves of control plant, indicated that *GhSNAP33* silencing triggered leaf cell death (**Figure 5D**). In addition, the second and third true leaves of *GhSNAP33*-deficient plants showed elevated reactive oxygen species (ROS) levels compared to control leaves, as determined by DAB staining (**Figure 5E**).

### *GhSNAP33*-Deficient Cotton Plants Are Susceptible to *V. dahliae* Infection

**FIGURE 6:**
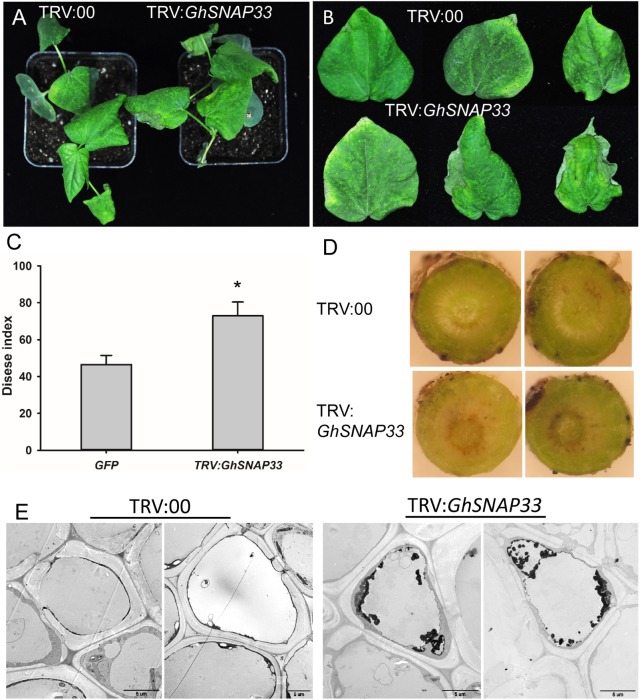
Susceptibility of *GhSNAP33*-deficient cotton plants to *V. dahliae* infection. **(A)** Disease symptoms of VIGS plants 12 dpi. **(B)** Representative leaves from plants at 12 dpi. **(C)** Disease index at 14 dpi. **(D)** Vascular browning in stems of plants infected with fungus. **(E)** The thylose in root cell of infected plants at 14 dpi. Data represent mean ± standard error of three independent repeats (*n* = 3) with at least 16 plants each. ^∗^*P* < 0.05 vs. control.

To analyze the role of *GhSNAP33* in the defense response of cotton against *V. dahliae*, *GhSNAP33*-silenced seedlings were inoculated with *V. dahliae*. Loss of *GhSNAP33* resulted in exacerbation of wilting relative to control plants (**Figure 6A**); the leaves became wilting along the edge of leaves at 12 day post inoculation (dpi) (**Figure 6B**). The disease index was also increased in these plants (**Figure 6C**). Additionally, deepened vascular browning (**Figure 6D**) and increased thylose accumulation (**Figure 6E**) in *GhSNAP33*-deficient plants confirmed their susceptibility to *V. dahliae* infection.

### *GhSNAP33* Overexpression Enhances *Arabidopsis* Resistance to *V. dahliae*

Hygromycin-resistant *GhSNAP33*-overexpressing *Arabidopsis* lines were identified by genomic PCR analysis (Supplementary Figure S3A). Homozygous transgenic (T3 generation) lines of L1 and L3 were selected for subsequent experiments based on semi-quantitative RT-PCR analysis (Supplementary Figure S3B).

**FIGURE 7:**
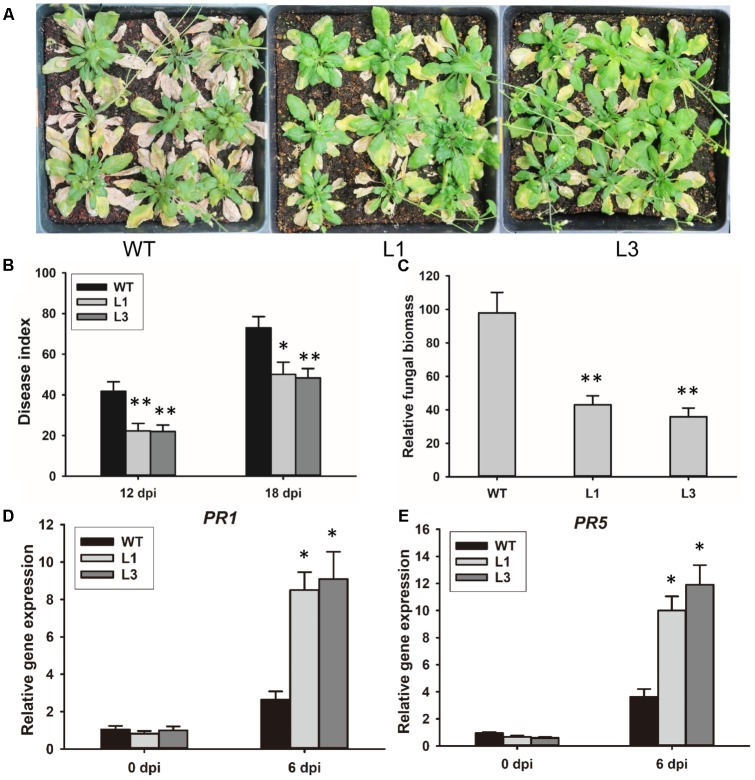
Resistance to *V. dahliae* infection in *Arabidopsis* conferred by *GhSNAP33* overexpression. **(A)** *Arabidopsis* plants were inoculated with *V. dahliae* by root dipping. Images were taken at 20 dpi. The assay was repeated three times. **(B)** Disease index of WT and transgenic plants at 12 and 18 dpi. **(C)** Biomass of *V. dahliae* in infected plants at 20 dpi. **(D)** The expression of *PR1* in infected plants. **(E)** The expression of *PR5* in infected plants. Data represent mean ± standard error of three independent repeats (*n* = 3). ^∗^*P* < 0.05, ^∗∗^*P* < 0.01 vs. WT.

To investigate the contribution of GhSNAP33 to disease resistance in plant, *GhSNAP33*-transgenic plants were inoculated with *V. dahliae* spores by the root dipping method. 3-week-old *GhSNAP33*-expressing *Arabidopsis* plants infected with *V. dahliae* showed apparently less wilt, yellowish and necrosis as compared to the WT at 20 dpi (**Figure 7A**); this was associated with a lower disease index (**Figure 7B**). An analysis of fungal biomass confirmed that there was less fungus in the two transgenic lines than in WT plants (**Figure 7C**) and the expression of *PR1* and *PR5* was increased in infected transgenic plants compared with infected WT plants (**Figures 7D,E**).

**FIGURE 8:**
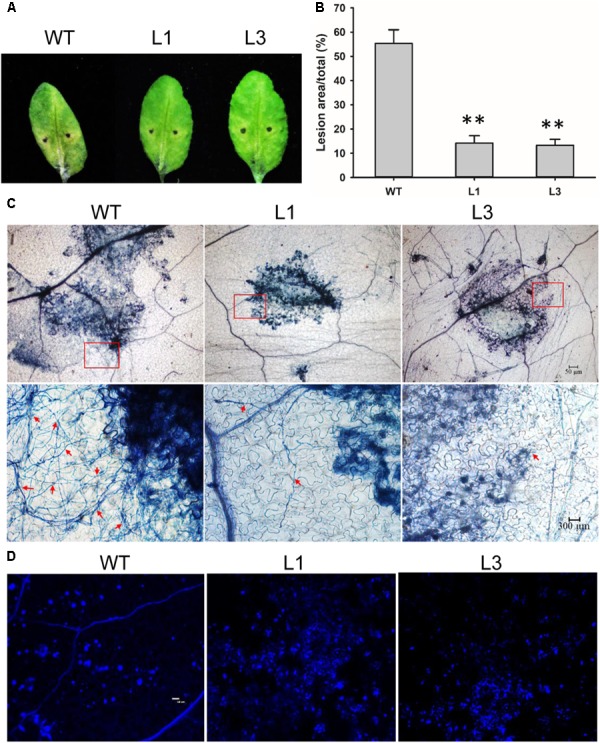
Elevated callose deposition and reduced mycelia growth in *GhSNAP33* transgenic *Arabidopsis*. **(A)** *V. dahliae* spores suspension was applied to detached WT and *GhSNAP33* transgenic plants; representative leaves are shown at 6 dpi. **(B)** Lesion area of infected plants at 6 dpi. **(C)** The mycelia growth and cell death on infected leaves at 6 dpi stained with trypan blue. Top panel: Trypan blue staining of leaves. Lower panel: Closeups from corresponding top panel. The fungal mycelia are indicated by red arrows. **(D)** Callose deposition on the infected leaves stained with aniline blue at 24 h after inoculation. Similar results were obtained in independent experiments. Data represent mean ± standard error of three independent repeats (*n* = 3). ^∗∗^*P* < 0.01 vs. WT.

Detached leaves of the *Arabidopsis* lines were inoculated with *V. dahliae* to evaluate the defense response. Chlorosis symptoms were milder in *V. dahliae*-infected leaves from transgenic as compared to WT plants at 6 dpi (**Figure 8A**). The fungal lesion area was smaller in the two transgenic line (**Figure 8B**). Trypan blue staining revealed that *V. dahliae* proliferated more quickly in leaves of WT plant with more mycelia growth extending the lesion area compared with transgenic plants L1 and L3 indicating *GhSNAP33* expression in *Arabidopsis* hindered *V. dahliae* mycelia growth (**Figure 8C**). Aniline blue staining showed enhanced callose deposition in *GhSNAP33*-transgenic plants (**Figure 8D**).

### *GhSNAP33* Overexpression Increases Plant Tolerance to Drought

**FIGURE 9:**
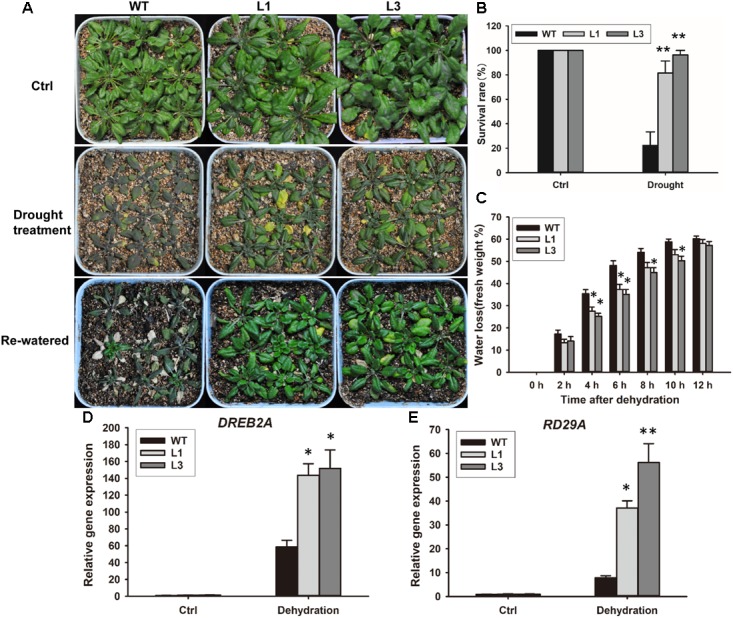
Enhanced plant drought tolerance in *Arabidopsis* by ectopic expression of *GhSNAP33*. **(A)** *GhSNAP33* overexpression reduced plant susceptibility to drought. Top panel: *Arabidopsis* plants under normal conditions. Middle panel: 3-week-old plants were subjected to water withholding for 20 days. Lower panel: plants after drought treatment were re-watered for 2 days. Experiments were repeated three times. **(B)** Survival rate of WT and *GhSNAP33*-overexpressing plants after a 2-day recovery from drought stress. **(C)** Reduced water loss in *GhSNAP33*-overexpressing plants. The weight of detached leaves was measured at indicated time points. **(D)** The expression of *DREB2A* in *Arabidopsis* plants after dehydration. **(E)** The expression of *RD29A* in *Arabidopsis* plants after dehydration. Data represent mean ± standard error of three independent repeats (*n* = 3). ^∗^*P* < 0.05, ^∗∗^*P* < 0.01 vs. WT.

Based on our observation that ABA and PEG600 induced *GhSNAP33* expression, we investigated the drought tolerance of *GhSNAP33*-transgenic *Arabidopsis* plants. The plants were in relatively good condition relative to their WT counterparts after 20 days without irrigation (**Figure 9A**). Most of the *GhSNAP33*-expressing plants recovered from drought after 2 days of re-watering (**Figure 9A**), accompanied with a higher survival rate than in WT (**Figure 9B**). Consistent with these findings, the dehydration assay showed that *GhSNAP33* overexpression decreased the rate of water loss in *Arabidopsis* (**Figure 9C**) and the expression of *DERB2A* and *RD29A* was increased in the both overexpression lines (**Figures 9D,E**).

## Discussion

SNAREs are essential for membrane fusion during vesicular transport in eukaryotic cells. SNAP25-type proteins regulate fusion between vesicles and plasma membrane during exo- and endocytosis in yeast and mammals. Of the 54 SNARE genes in the *Arabidopsis* genome, three encode SNAP25-type proteins. One of these, AtSNAP33, localizes to the plasma membrane and functions in exocytosis during cell division and in the plant defense response (Sanderfoot et al., 2000; Uemura et al., 2004). Although several SNAP25-type genes have been cloned in plants, there have been no reports on cotton SNARE family proteins. In the present study, we characterized *GhSNAP33*, a SNAP25-type t-SNARE gene, from cotton. GhSNAP33 contains C- and N-terminal Qb- and Qc-SNARE motifs, respectively, which are evolutionarily conserved stretches of 60–70 amino acids arranged as heptad repeats (Jahn and Scheller, 2006). These motifs are connected by an anti-parallel linker that anchors the protein to the cell membrane and contributes to exocytosis in mammals (Gonzalo et al., 1999; Nagy et al., 2008). The GhSNAP33-GFP protein in transgenic *Arabidopsis* was expressed at the plasma membrane (Supplementary Figure S2), consistent with the subcellular localization of AtSNAP33 (Heese et al., 2001), OsSNAP32 (Bao et al., 2008b), and CkSNAP33 (Wang et al., 2017). The amino acid sequence of GhSNAP33 shared high sequence identity with other SNAP25-type proteins, including CkSNAP33 from *C. komarovii* (69.51%), GsSNAP33 (67.54%) *A. thaliana* AtSNAP33 (63.61%), *Oryza sativa* L. OsSNAP32 (53.44%), and *Hordeum vulgare* L. HvSNAP34 (52.38%), indicating they may have similar functions in plant development and defense response to abiotic and biotic stress.

SNAP25-type proteins are essential for growth and development in all organisms. In mammals, loss of function of a key SNAP25-type protein leads to physical defects and disease (Rapaport et al., 2010; Browning and Karim, 2013). AtSNAP33 is ubiquitously expressed in roots, stems, leaves, and flowers of *Arabidopsis* and the *atsnap33* mutant developed large necrotic lesions on cotyledons and rosette leaves, and died before flowering (Heese et al., 2001). *StSNAP33*-deficient potato showed high levels of free SA at 3 weeks and exhibited spontaneous necrosis and chlorosis at later stages (Eschen-Lippold et al., 2012). A tissue-specific analysis of *GhSNAP33* revealed higher expression in roots and leaves than in the stem of cotton plants. *GhSNAP33* knockdown yielded smaller plants and spontaneous lesion on the senior true leaf, which was associated with increased cell death and ROS accumulation. These results suggest that *GhSNAP33* is critical for cotton development, although further study is needed to determine whether the phenotypic defect is related to compromised cytokinesis resulting from the absence of *GhSNAP33*.

The SNARE-mediated secretory pathway delivers cellular defense factors to infection sites during exocytosis-associated immune responses in plants (Kwon et al., 2008a). SNAP25-type proteins have been shown to contribute to disease resistance in plants: for example, HvSNAP34 against powdery mildew (*Blumeria graminis* f. sp. *Hordei*) in barley (Collins et al., 2003), OsSNAP32 against blast fungus (Luo et al., 2016), and CkSNAP33 against *V. dahliae* infection (Wang et al., 2017). *GhSNAP33* transcription was upregulated in response to ABA, SA and H_2_O_2_ treatment and *V. dahliae* infection, and *GhSNAP33*-deficient plants were more susceptible to *V. dahliae*, as evidenced by the greater severity of disease symptoms, elevated disease index, deepened vascular browning and increased thylose accumulation. On the other hand, *GhSNAP33* overexpression in *Arabidopsis* enhanced disease resistance relative to WT plants, accompanied with elevated expression of *PR1* and *PR5*; the results of the fungal filtrate assay revealed that leaves from transgenic plants had less chlorosis and fungal mycelia, and more callose deposition providing further evidence for the involvement of GhSNAP33 in disease resistance against *V. dahliae.*

With the changing of global climate, drought stress is becoming a major environmental problem affecting crop growth, development, and production (Ahuja et al., 2010). GsSNAP33, the *G. soja* homolog of GhSNAP33, is involved in plant tolerance to drought and salt stress in *Arabidopsis* (Nisa et al., 2017). We found that *GhSNAP33* overexpression was enhanced by ABA and PEG6000 treatments and ectopic expression of *GhSNAP33* increased yeast tolerance to mannitol. Importantly, transgenic *Arabidopsis* plants expressing *GhSNAP33* showed heightened tolerance to drought as compared to the WT with high survival rate after drought treatment. These results were supported by the observation that transgenic plants exhibited reduced rates of water loss and elevated expression of drought-responsive genes, *DREB2A* and *RD29A*, under conditions of dehydration.

The upregulation of *GhSNAP33* expression upon ABA and SA treatments indicates that GhSNAP33 may be involved in the cotton hormone-mediated signaling pathways. It has been reported that the systemic induction of *AtSNAP33* is SA dependent (Wick et al., 2003) indicating the implication of SNAP25-type protein in SA signaling pathway. SNAREs have been implicated in ABA-mediated responses to abiotic stress (Carter et al., 2004) and to pathogen resistance (Collins et al., 2003). Therefore, GhSNAP33 may implicate in ABA-mediated drought responses and ABA-dependent callose deposition after *V. dahliae* infection. In view of the antagonism of SA-dependent resistance by ABA in plant–pathogen interaction (Ton et al., 2009), further work is needed to make sense of the effect of GhSNAP33 on SA and ABA signaling pathway and interplay between ABA- and SA-dependent defense pathway in cotton stress response.

In pathogen–plant interactions, the SNARE complex mediates immune responses through focal secretion (Bednarek et al., 2010; Yun and Kwon, 2012). In addition to pathogenesis-related (PR) proteins, secondary cell wall components and/or enzymes may be transported to achieve plant immunity (Collins et al., 2003; Assaad et al., 2004; Kalde et al., 2007; Yun and Kwon, 2017). SNAP25-type proteins catalyze vesicle exocytosis by forming a ternary SNARE complex with other two other SNARE family members containing Qa- and R-SNARE motifs. The PEN1/SYNTAXIN OF PLANTS (SYP) 122/SYP132–SNAP33–VAMP721/722 pathway is a default secretory pathway essential for growth and development and the defense response in plants (Bednarek et al., 2010). SNAP33, as the only SNAP25-type protein in these processes, is essential for the focal secretion. GhSNAP33 may play similar roles in the defense response. The enhanced callose deposition in *GhSNAP33* transgenic plants suggested that GhSNAP33 may also be involved in the directional delivery of callose precursors and/or the callose synthase-like protein to infection sites, since the secretory PEN1–SNAP33–VAMP721/722 complex is essential for the penetration resistance of cell wall at early time points in *Arabidopsis* (Kwon et al., 2008a; Bednarek et al., 2010). NbSYP132 has been reported was implicated in the exocytosis of vesicles containing antimicrobial PR1 (Kalde et al., 2007). The elevated expression of *PR1* and *PR5* in *GhSNAP33* transgenic plants may be related to the exocytosis mediated by cognate SNARE complex. SNARE proteins also participate in tip-focused membrane trafficking for root hair tip growth in *Arabidopsis* (Ichikawa et al., 2014) and in trafficking of plasma membrane Aquaporin for the modulation of cell membrane water permeability (Besserer et al., 2012; Hachez et al., 2014). It is possible that GhSNAP33 is involved in a SNARE complex that transports similar factors and affects the drought-related maker genes including *DREB2A* and *RD29A* to mediate drought tolerance in cotton.

In summary, our functional analysis of GhSNAP33, a synaptosome-associated t-SNARE protein, revealed a potential role in vesicle trafficking in cotton development and defense responses. Loss of *GhSNAP33* expression resulted in cell death and increased ROS production in cotton leaves, and compromised plant resistance against *V. dahliae* infection. Conversely, resistance to *V. dahliae* was enhanced by *GhSNAP33* overexpression. Ectopic expression of *GhSNAP33* increased tolerance to osmotic stress in yeast and drought tolerance in *Arabidopsis*. Thus, GhSNAP33 is not only essential for the development of cotton plants but is critical for plant drought tolerance and resistance to *V. dahliae*. These findings provide a basis for developing strategies to improve drought tolerance and disease resistance in cotton plants to meet emerging environmental challenges. However, additional research is needed to clarify the role of GhSNAP33 in the specific secret pathway via membrane fusion in cotton.

## Author Contributions

PW, FL, and YH conceived and designed the study. PW, YS, and YP conducted the experiments. PW, XL, and XZ performed the data analysis. PW and YH drafted the manuscript. All authors were reviewed and revised the manuscript and figures.

## Conflict of Interest Statement

The authors declare that the research was conducted in the absence of any commercial or financial relationships that could be construed as a potential conflict of interest.

## Footnotes

1 http://web.expasy.org/protparam/

2 http://www.cbs.dtu.dk/services/TMHMM-2.0

3 http://www.ebi.ac.uk/Tools/msa/clustalo/

4 http://www.ch.embnet.org/software/BOX_form.html

5 https://www.ncbi.nlm.nih.gov/Structure/cdd/wrpsb.cgi

## References

[B1] Ahuja I. De Vos R. C. Bones A. M. Hall R. D. (2010). Plant molecular stress responses face climate change. *Trends Plant Sci.* 15 664–674. 10.1016/j.tplants.2010.08.002 20846898

[B2] An Y. Wang Y. Lou L. Zheng T. Qu G. Z. (2011). A novel zinc-finger-like gene from *Tamarix hispida* is involved in salt and osmotic tolerance. *J. Plant Res.* 124 689–697. 10.1007/s10265-011-0403-4 21327695

[B3] Ashraf J. Zuo D. Wang Q. Malik W. Zhang Y. Abid M. A. (2018). Recent insights into cotton functional genomics: progress and future perspectives. *Plant Biotechnol. J.* 16 699–713. 10.1111/pbi.12856 29087016 PMC5814580

[B4] Assaad F. F. Qiu J. L. Youngs H. Ehrhardt D. Zimmerli L. Kalde M. (2004). The PEN1 syntaxin defines a novel cellular compartment upon fungal attack and is required for the timely assembly of papillae. *Mol. Biol. Cell* 15 5118–5129. 10.1091/mbc.e04-02-0140 15342780 PMC524786

[B5] Bao Y. M. Wang J. F. Huang J. Zhang H. S. (2008a). Cloning and characterization of three genes encoding Qb-SNARE proteins in rice. *Mol. Genet. Genomics* 279 291–301. 10.1007/s00438-007-0313-2 18197419

[B6] Bao Y. M. Wang J. F. Huang J. Zhang H. S. (2008b). Molecular cloning and characterization of a novel SNAP25-type protein gene *OsSNAP32* in rice (*Oryza sativa* L.). *Mol. Biol. Rep.* 35 145–152. 17380428 10.1007/s11033-007-9064-8

[B7] Bassham D. C. Blatt M. R. (2008). SNAREs: cogs and coordinators in signaling and development. *Plant Physiol.* 147 1504–1515. 10.1104/pp.108.121129 18678742 PMC2492632

[B8] Bednarek P. Kwon C. Schulze-Lefert P. (2010). Not a peripheral issue: secretion in plant-microbe interactions. *Curr. Opin. Plant Biol.* 13 378–387. 10.1016/j.pbi.2010.05.002 20558098

[B9] Besserer A. Burnotte E. Bienert G. P. Chevalier A. S. Errachid A. Grefen C. (2012). Selective regulation of maize plasma membrane aquaporin trafficking and activity by the SNARE SYP121. *Plant Cell* 24 3463–3481. 10.1105/tpc.112.101758 22942383 PMC3462644

[B10] Bock J. B. Matern H. T. Peden A. A. Scheller R. H. (2001). A genomic perspective on membrane compartment organization. *Nature* 409 839–841. 10.1038/35057024 11237004

[B11] Bonifacino J. S. Glick B. S. (2004). The mechanisms of vesicle budding and fusion. *Cell* 116 153–166. 10.1016/S0092-8674(03)01079-1 14744428

[B12] Browning R. Karim S. (2013). RNA interference-mediated depletion of N-ethylmaleimide sensitive fusion protein and synaptosomal associated protein of 25 kDa results in the inhibition of blood feeding of the Gulf Coast tick, *Amblyomma maculatum*. *Insect Mol. Biol.* 22 245–257. 10.1111/imb.12017 23437815 PMC3644323

[B13] Carter C. J. Bednarek S. Y. Raikhel N. V. (2004). Membrane trafficking in plants: new discoveries and approaches. *Curr. Opin. Plant Biol.* 7 701–707. 10.1016/j.pbi.2004.09.016 15491919

[B14] Chen J. Li N. Ma X. Gupta V. K. Zhang D. Li T. (2017). The ectopic overexpression of the cotton *Ve1* and *Ve2*-homolog sequences leads to resistance response to *Verticillium* wilt in *Arabidopsis*. *Front. Plant Sci.* 8:844. 10.3389/fpls.2017.00844 28611793 PMC5447073

[B15] Chen T. Kan J. Yang Y. Ling X. Chang Y. Zhang B. (2016). A Ve homologous gene from *Gossypium barbadense*, *Gbvdr3*, enhances the defense response against *Verticillium dahliae*. *Plant Physiol. Biochem.* 98 101–111. 10.1016/j.plaphy.2015.11.015 26686282

[B16] Collins N. C. Thordal-Christensen H. Lipka V. Bau S. Kombrink E. Qiu J. L. (2003). SNARE-protein-mediated disease resistance at the plant cell wall. *Nature* 425 973–977. 10.1038/nature02076 14586469

[B17] Cox K. L. Meng F. Wilkins K. E. Li F. Wang P. Booher N. J. (2017). TAL effector driven induction of a *SWEET* gene confers susceptibility to bacterial blight of cotton. *Nat. Commun.* 8:15588. 10.1038/ncomms15588 28537271 PMC5458083

[B18] Daayf F. Nicole M. Geiger J. P. (1995). Differentiation of *Verticillium dahliae* populations on the basis of vegetative compatibility and pathogenicity on cotton. *Eur. J. Plant Pathol.* 101 69–79. 10.1007/BF01876095

[B19] Dong Y. Burch-Smith T. M. Liu Y. Mamillapalli P. Dinesh-Kumar S. P. (2007). A ligation-independent cloning tobacco rattle virus vector for high-throughput virus-induced gene silencing identifies roles for *NbMADS4-1* and *-2* in floral development. *Plant Physiol.* 145 1161–1170. 10.1104/pp.107.107391 17932306 PMC2151726

[B20] Ernst J. A. Brunger A. T. (2003). High resolution structure, stability, and synaptotagmin binding of a truncated neuronal SNARE complex. *J. Biol. Chem.* 278 8630–8636. 10.1074/jbc.M211889200 12496247

[B21] Eschen-Lippold L. Landgraf R. Smolka U. Schulze S. Heilmann M. Heilmann I. (2012). Activation of defense against *Phytophthora infestans* in potato by down-regulation of syntaxin gene expression. *New Phytol.* 193 985–996. 10.1111/j.1469-8137.2011.04024.x 22243492

[B22] Fradin E. F. Thomma B. P. (2006). Physiology and molecular aspects of *Verticillium* wilt diseases caused by *V. dahliae* and *V. albo-atrum*. *Mol. Plant Pathol.* 7 71–86. 10.1111/j.1364-3703.2006.00323.x 20507429

[B23] Fukuda R. Mcnew J. A. Weber T. Parlati F. Engel T. Nickel W. (2000). Functional architecture of an intracellular membrane t-SNARE. *Nature* 407 198–202. 10.1038/35025084 11001059

[B24] Gao W. Long L. Zhu L. F. Xu L. Gao W. H. Sun L. Q. (2013). Proteomic and virus-induced gene silencing (VIGS) Analyses reveal that gossypol, brassinosteroids, and jasmonic acid contribute to the resistance of cotton to *Verticillium dahliae*. *Mol. Cell. Proteomics* 12 3690–3703. 10.1074/mcp.M113.031013 24019146 PMC3861717

[B25] Gao X. Li F. Li M. Kianinejad A. S. Dever J. K. Wheeler T. A. (2013). Cotton *GhBAK1* mediates *Verticillium* wilt resistance and cell death. *J. Integr. Plant Biol.* 55 586–596. 10.1111/jipb.12064 23675706 PMC4395461

[B26] Gao X. Wheeler T. Li Z. Kenerley C. M. He P. Shan L. (2011). Silencing *GhNDR1* and *GhMKK2* compromises cotton resistance to *Verticillium* wilt. *Plant J.* 66 293–305. 10.1111/j.1365-313X.2011.04491.x 21219508 PMC3078967

[B27] Garas N. A. Wilhem S. Sagen J. E. (1986). Relationship of cultivar resistance to distribution of *Verticillium dahliae* in inoculated cotton plants and to growth of single conidia on excised stem segments. *Phytopathology* 76 1005–1010. 10.1094/Phyto-76-1005

[B28] Gonzalo S. Greentree W. K. Linder M. E. (1999). SNAP-25 is targeted to the plasma membrane through a novel membrane-binding domain. *J. Biol. Chem.* 274 21313–21318. 10.1074/jbc.274.30.21313 10409690

[B29] Gu Z. Liu T. Ding B. Li F. Wang Q. Qian S. (2017). Two lysin-motif receptor kinases, Gh-LYK1 and Gh-LYK2, contribute to resistance against *Verticillium* wilt in upland cotton. *Front. Plant Sci.* 8:2133. 10.3389/fpls.2017.02133 29326741 PMC5733346

[B30] Guo Y. Pang C. Jia X. Ma Q. Dou L. Zhao F. (2017). An NAM domain gene, *GhNAC79*, improves resistance to drought stress in upland cotton. *Front. Plant Sci.* 8:1657. 10.3389/fpls.2017.01657 28993786 PMC5622203

[B31] Hachez C. Laloux T. Reinhardt H. Cavez D. Degand H. Grefen C. (2014). *Arabidopsis* SNAREs SYP61 and SYP121 coordinate the trafficking of plasma membrane aquaporin PIP2;7 to modulate the cell membrane water permeability. *Plant Cell* 26 3132–3147. 10.1105/tpc.114.127159 25082856 PMC4145137

[B32] Heese M. Gansel X. Sticher L. Wick P. Grebe M. Granier F. (2001). Functional characterization of the KNOLLE-interacting t-SNARE AtSNAP33 and its role in plant cytokinesis. *J. Cell Biol.* 155 239–249. 10.1083/jcb.200107126 11591731 PMC2198836

[B33] Hill M. K. Lyon K. J. Lyon B. R. (1999). Identification of disease response genes expressed in *Gossypium hirsutum* upon infection with the wilt pathogen *Verticillium dahliae*. *Plant Mol. Biol.* 40 289–296. 10.1023/A:1006146419544 10412907

[B34] Holt M. Varoqueaux F. Wiederhold K. Takamori S. Urlaub H. Fasshauer D. (2006). Identification of SNAP-47, a novel Qbc-SNARE with ubiquitous expression. *J. Biol. Chem.* 281 17076–17083. 10.1074/jbc.M513838200 16621800

[B35] Ichikawa M. Hirano T. Enami K. Fuselier T. Kato N. Kwon C. (2014). Syntaxin of plant proteins SYP123 and SYP132 mediate root hair tip growth in *Arabidopsis thaliana*. *Plant Cell Physiol.* 55 790–800. 10.1093/pcp/pcu048 24642714

[B36] Jahn R. Scheller R. H. (2006). SNAREs-engines for membrane fusion. *Nat. Rev. Mol. Cell Biol.* 7 631–643. 10.1038/nrm2002 16912714

[B37] Kalde M. Nuhse T. S. Findlay K. Peck S. C. (2007). The syntaxin SYP132 contributes to plant resistance against bacteria and secretion of pathogenesis-related protein 1. *Proc. Natl Acad. Sci. U.S.A.* 104 11850–11855. 10.1073/pnas.0701083104 17592123 PMC1913864

[B38] Kerr T. C. Abdel-Mageed H. Aleman L. Lee J. Payton P. Cryer D. (2017). Ectopic expression of two AREB/ABF orthologs increases drought tolerance in cotton (*Gossypium hirsutum*). *Plant Cell Environ.* 41 898–907. 10.1111/pce.12906 28098349

[B39] Klosterman S. J. Atallah Z. K. Vallad G. E. Subbarao K. V. (2009). Diversity, pathogenicity, and management of *Verticillium* species. *Annu. Rev. Phytopathol.* 47 39–62. 10.1146/annurev-phyto-080508-081748 19385730

[B40] Kwon C. Bednarek P. Schulze-Lefert P. (2008a). Secretory pathways in plant immune responses. *Plant Physiol.* 147 1575–1583. 10.1104/pp.108.121566 18678749 PMC2492620

[B41] Kwon C. Neu C. Pajonk S. Yun H. S. Lipka U. Humphry M. (2008b). Co-option of a default secretory pathway for plant immune responses. *Nature* 451 835–840. 10.1038/nature06545 18273019

[B42] Lee J. H. Van Montagu M. Verbruggen N. (1999). A highly conserved kinase is an essential component for stress tolerance in yeast and plant cells. *Proc. Natl Acad. Sci. U.S.A.* 96 5873–5877. 10.1073/pnas.96.10.5873 10318977 PMC21953

[B43] Li F. Fan G. Lu C. Xiao G. Zou C. Kohel R. J. (2015). Genome sequence of cultivated Upland cotton (*Gossypium hirsutum* TM-1) provides insights into genome evolution. *Nat. Biotechnol.* 33 524–530. 10.1038/nbt.3208 25893780

[B44] Li F. Fan G. Wang K. Sun F. Yuan Y. Song G. (2014). Genome sequence of the cultivated cotton *Gossypium arboreum*. *Nat. Genet.* 46 567–572. 10.1038/ng.2987 24836287

[B45] Li F. Li M. Wang P. Cox KL Jr Duan L. Dever J. K. (2017). Regulation of cotton (*Gossypium hirsutum*) drought responses by mitogen-activated protein (MAP) kinase cascade-mediated phosphorylation of GhWRKY59. *New Phytol.* 215 1462–1475. 10.1111/nph.14680 28700082

[B46] Liu N. Ma X. Zhou S. Wang P. Sun Y. Li X. (2016). Molecular and functional characterization of a polygalacturonase-inhibiting protein from *Cynanchum komarovii* that confers fungal resistance in *Arabidopsis*. *PLoS One* 11:e0146959. 10.1371/journal.pone.0146959 26752638 PMC4709088

[B47] Liu N. Sun Y. Pei Y. Zhang X. Wang P. Li X. (2018). A pectin methylesterase inhibitor enhances resistance to *Verticillium* wilt. *Plant Physiol.* 176 2202–2220. 10.1104/pp.17.01399 29363564 PMC5841709

[B48] Liu N. Zhang X. Sun Y. Wang P. Li X. Pei Y. (2017). Molecular evidence for the involvement of a polygalacturonase-inhibiting protein, GhPGIP1, in enhanced resistance to *Verticillium* and Fusarium wilts in cotton. *Sci. Rep.* 7:39840. 10.1038/srep39840 28079053 PMC5228132

[B49] Liu W. X. Zhang F. C. Zhang W. Z. Song L. F. Wu W. H. Chen Y. F. (2013). *Arabidopsis* Di19 functions as a transcription factor and modulates *PR1*, *PR2*, and *PR5* expression in response to drought stress. *Mol. Plant* 6 1487–1502. 10.1093/mp/sst031 23404561

[B50] Luo J. Zhang H. He W. W. Zhang Y. Cao W. L. Zhang H. S. (2016). *OsSNAP32*, a SNAP25-type SNARE protein-encoding gene from rice, enhanced resistance to blast fungus. *Plant Growth Regul.* 80 37–45. 10.1007/s10725-016-0152-4

[B51] Ma L. F. Hu L. X. Fan J. B. Amombo E. Khaldun A. B. M. Zheng Y. (2017). Cotton *GhERF38* gene is involved in plant response to salt/drought and ABA. *Ecotoxicology* 26 841–854. 10.1007/s10646-017-1815-2 28536792

[B52] McNew J. A. Parlati F. Fukuda R. Johnston R. J. Paz K. Paumet F. (2000). Compartmental specificity of cellular membrane fusion encoded in SNARE proteins. *Nature* 407 153–159. 10.1038/35025000 11001046

[B53] Meyer D. Pajonk S. Micali C. O’connell R. Schulze-Lefert P. (2009). Extracellular transport and integration of plant secretory proteins into pathogen-induced cell wall compartments. *Plant J.* 57 986–999. 10.1111/j.1365-313X.2008.03743.x 19000165

[B54] Mishra N. Sun L. Zhu X. Smith J. Prakash Srivastava A. Yang X. (2017). Overexpression of the rice SUMO E3 Ligase Gene *OsSIZ1* in cotton enhances drought and heat tolerance, and substantially improves fiber yields in the field under reduced irrigation and rainfed conditions. *Plant Cell Physiol.* 58 735–746. 10.1093/pcp/pcx032 28340002 PMC5444567

[B55] Nagy G. Milosevic I. Mohrmann R. Wiederhold K. Walter A. M. Sorensen J. B. (2008). The SNAP-25 linker as an adaptation toward fast exocytosis. *Mol. Biol. Cell* 19 3769–3781. 10.1091/mbc.E07-12-1218 18579690 PMC2526689

[B56] Nisa Z. U. Mallano A. I. Yu Y. Chen C. Duan X. Amanullah S. (2017). GsSNAP33 a novel *Glycine soja* SNAP25-type protein gene: Improvement of plant salt and drought tolerances in transgenic *Arabidopsis thaliana. Plant Physiol. Biochem.* 119 9–20. 10.1016/j.plaphy.2017.07.029 28841544

[B57] Qu Z. L. Wang H. Y. Xia G. X. (2005). GhHb1: a nonsymbiotic hemoglobin gene of cotton responsive to infection by *Verticillium dahliae*. *Biochim. Biophys. Acta* 1730 103–113. 10.1016/j.bbaexp.2005.06.009 16084605

[B58] Rapaport D. Lugassy Y. Sprecher E. Horowitz M. (2010). Loss of SNAP29 impairs endocytic recycling and cell motility. *PLoS One* 5:e9759. 10.1371/journal.pone.0009759 20305790 PMC2841205

[B59] Sakurai C. Hashimoto H. Nakanishi H. Arai S. Wada Y. Sun-Wada G. H. (2012). SNAP-23 regulates phagosome formation and maturation in macrophages. *Mol. Biol. Cell* 23 4849–4863. 10.1091/mbc.E12-01-0069 23087210 PMC3521691

[B60] Sanderfoot A. A. Assaad F. F. Raikhel N. V. (2000). The Arabidopsis genome. An abundance of soluble N-ethylmaleimide-sensitive factor adaptor protein receptors. *Plant Physiol.* 124 1558–1569. 10.1104/pp.124.4.1558 11115874 PMC59855

[B61] Schilde C. Lutter K. Kissmehl R. Plattner H. (2008). Molecular identification of a SNAP-25-like SNARE protein in *Paramecium*. *Eukaryot. Cell* 7 1387–1402. 10.1128/EC.00012-08 18552286 PMC2519768

[B62] Shi H. Ye T. Chen F. Cheng Z. Wang Y. Yang P. (2013). Manipulation of arginase expression modulates abiotic stress tolerance in *Arabidopsis*: effect on arginine metabolism and ROS accumulation. *J. Exp. Bot.* 64 1367–1379. 10.1093/jxb/ers400 23378380 PMC3598423

[B63] Steegmaier M. Yang B. Yoo J. S. Huang B. Shen M. Yu S. (1998). Three novel proteins of the syntaxin/SNAP-25 family. *J. Biol. Chem.* 273 34171–34179. 10.1074/jbc.273.51.34171 9852078

[B64] Strop P. Kaiser S. E. Vrljic M. Brunger A. T. (2008). The structure of the yeast plasma membrane SNARE complex reveals destabilizing water-filled cavities. *J. Biol. Chem.* 283 1113–1119. 10.1074/jbc.M707912200 17956869

[B65] Sunilkumar G. Campbell L. M. Puckhaber L. Stipanovic R. D. Rathore K. S. (2006). Engineering cottonseed for use in human nutrition by tissue-specific reduction of toxic gossypol. *Proc. Natl Acad. Sci. U.S.A.* 103 18054–18059. 10.1073/pnas.0605389103 17110445 PMC1838705

[B66] Thatcher L. F. Manners J. M. Kazan K. (2009). Fusarium oxysporum hijacks COI1-mediated jasmonate signaling to promote disease development in *Arabidopsis. Plant J.* 58 927–939. 10.1111/j.1365-313X.2009.03831.x 19220788

[B67] Ton J. Flors V. Mauch-Mani B. (2009). The multifaceted role of ABA in disease resistance. *Trends Plant Sci.* 14 310–317. 10.1016/j.tplants.2009.03.006 19443266

[B68] Uemura T. Ueda T. Ohniwa R. L. Nakano A. Takeyasu K. Sato M. H. (2004). Systematic analysis of SNARE molecules in Arabidopsis: dissection of the post-Golgi network in plant cells. *Cell Struct. Funct.* 29 49–65. 10.1247/csf.29.49 15342965

[B69] Wang F. X. Ma Y. P. Yang C. L. Zhao P. M. Yao Y. Jian G. L. (2011a). Proteomic analysis of the sea-island cotton roots infected by wilt pathogen *Verticillium dahliae*. *Proteomics* 11 4296–4309. 10.1002/pmic.201100062 21928292

[B70] Wang Q. Zhang X. Li F. Hou Y. Liu X. Zhang X. (2011b). Identification of a UDP-glucose pyrophosphorylase from cotton (*Gossypium hirsutum* L.) involved in cellulose biosynthesis in *Arabidopsis thaliana*. *Plant Cell Rep.* 30 1303–1312. 10.1007/s00299-011-1042-x 21373794

[B71] Wang K. Wang Z. Li F. Ye W. Wang J. Song G. (2012). The draft genome of a diploid cotton *Gossypium raimondii*. *Nat. Genet.* 44 1098–1103. 10.1038/ng.2371 22922876

[B72] Wang L. Bittner M. A. Axelrod D. Holz R. W. (2008). The structural and functional implications of linked SNARE motifs in SNAP25. *Mol. Biol. Cell* 19 3944–3955. 10.1091/mbc.E08-04-0344 18596234 PMC2526714

[B73] Wang P. Zhang X. Ma X. Sun Y. Liu N. Li F. (2017). Identification of *CkSNAP33*, a gene encoding synaptosomal-associated protein from *Cynanchum komarovii*, that enhances *Arabidopsis* resistance to *Verticillium dahliae*. *PLoS One* 12:e0178101. 10.1371/journal.pone.0178101 28575006 PMC5456056

[B74] Wick P. Gansel X. Oulevey C. Page V. Studer I. Durst M. (2003). The expression of the t-SNARE AtSNAP33 is induced by pathogens and mechanical stimulation. *Plant Physiol.* 132 343–351. 10.1104/pp.102.012633 12746539 PMC166979

[B75] Xiao S. Y. Brown S. Patrick E. Brearley C. Turner J. G. (2003). Enhanced transcription of the *Arabidopsis* disease resistance genes *RPW8.1 and RPW8.2* via a salicylic acid-dependent amplification circuit is required for hypersensitive cell death. *Plant Cell* 15 33–45. 10.1105/tpc.006940 12509520 PMC143449

[B76] Yang C. Guo W. Li G. Gao F. Lin S. Zhang T. (2008). QTLs mapping for *Verticillium* wilt resistance at seedling and maturity stages in *Gossypium barbadense* L. *Plant Sci.* 174 290–298. 10.1016/j.plantsci.2007.11.016

[B77] Yun H. S. Kang B. G. Kwon C. (2016). *Arabidopsis* immune secretory pathways to powdery mildew fungi. *Plant Signal. Behav.* 11:e1226456. 10.1080/15592324.2016.1226456 27562527 PMC5257168

[B78] Yun H. S. Kwon C. (2012). Trafficking at the host cell surface during plant immune responses. *J. Plant Biol.* 55 185–190. 10.1007/s12374-011-0411-x

[B79] Yun H. S. Kwon C. (2017). Vesicle trafficking in plant immunity. *Curr. Opin. Plant Biol.* 40 34–42. 10.1016/j.pbi.2017.07.001 28735164

[B80] Zahoor R. Zhao W. Abid M. Dong H. Zhou Z. (2017). Potassium application regulates nitrogen metabolism and osmotic adjustment in cotton (*Gossypium hirsutum* L.) functional leaf under drought stress. *J. Plant Physiol.* 215 30–38. 10.1016/j.jplph.2017.05.001 28527336

[B81] Zhang B. Yang Y. Chen T. Yu W. Liu T. Li H. (2012). Island cotton *Gbve1* gene encoding a receptor-like protein confers resistance to both defoliating and non-defoliating isolates of *Verticillium dahliae*. *PLoS One* 7:e51091. 10.1371/journal.pone.0051091 23251427 PMC3519487

[B82] Zhang F. Li S. Yang S. Wang L. Guo W. (2015). Overexpression of a cotton annexin gene, GhAnn1, enhances drought and salt stress tolerance in transgenic cotton. *Plant Mol. Biol.* 87 47–67. 10.1007/s11103-014-0260-3 25330941

[B83] Zhang L. Wang M. Li N. Wang H. Qiu P. Pei L. (2017). Long noncoding RNAs involve in resistance to *Verticillium dahliae*, a fungal disease in cotton. *Plant Biotechnol. J.* 16 1172–1185. 10.1111/pbi.12861 29149461 PMC5978870

[B84] Zhang T. Hu Y. Jiang W. Fang L. Guan X. Chen J. (2015). Sequencing of allotetraploid cotton (*Gossypium hirsutum* L. acc. TM-1) provides a resource for fiber improvement. *Nat. Biotechnol.* 33 531–537. 10.1038/nbt.3207 25893781

[B85] Zhao F. Fang W. Xie D. Zhao Y. Tang Z. Li W. (2012). Proteomic identification of differentially expressed proteins in *Gossypium thurberi* inoculated with cotton *Verticillium dahliae*. *Plant Sci.* 18 176–184. 10.1016/j.plantsci.2011.10.007 22325879

