# Supplementary material for: GhSNAP33, a t-SNARE Protein From Gossypium hirsutum, Mediates Resistance to Verticillium dahliae Infection and Tolerance to Drought Stress
Source: Front Plant Sci. 2018 Jul 3;9:896. doi: 10.3389/fpls.2018.00896 (PMC6038728; doi:10.3389/fpls.2018.00896)
Supplement: Supplementary file 1 [file Data_Sheet_1.DOCX]

***Supplementary material***

**GhSNAP33, a t-SNARE protein from *Gossypium hirsutum*, mediates resistance to *Verticillium dahliae* infection and tolerance to drought stress**

**Ping Wang^1^, Yun Sun^1^, Yakun Pei^1^, Xiancai Li^1^, Xueyan Zhang^2^, Fuguang Li^2^ *, Yuxia Hou^1^ ***

*** Correspondence:** Fuguang Li: aylifug@163.com;

Yuxia Hou: houyuxia@cau.edu.cn;

**Supplementary Figures**

**
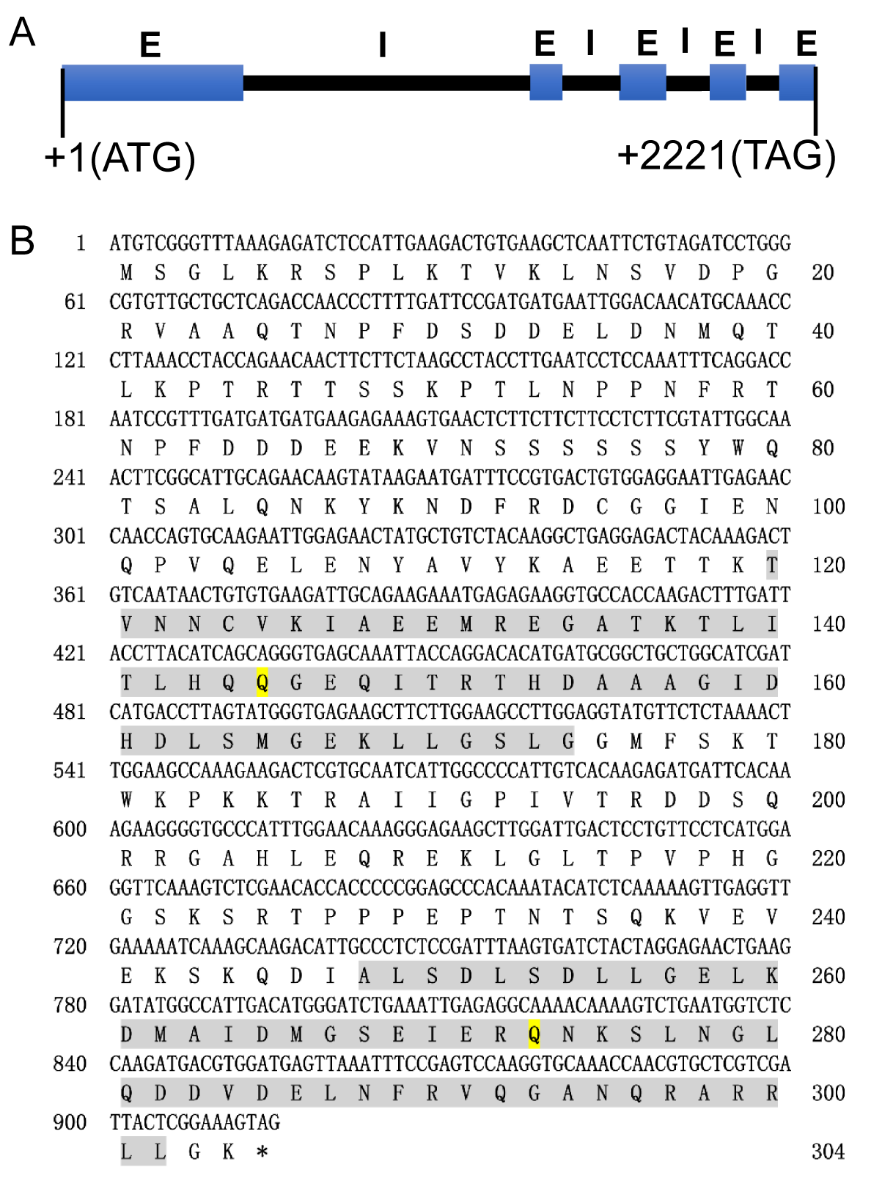
**

**Supplementary Figure 1. The schematic structure and sequence of *GhSNAP33*. (**A) Schematic representation of the *GhSNAP33* genomic DNA. Exon (E) and intron (I) are shaded in blue and black, respectively. (B) Nuclotide sequence and deduced amino acid sequence of *GhSNAP33.* The Qb-SNARE and Qc-SNARE domains are in gray shadow. The amino acids in yellow shadow are the conserved glutamine residues of Q-SNARE domain.


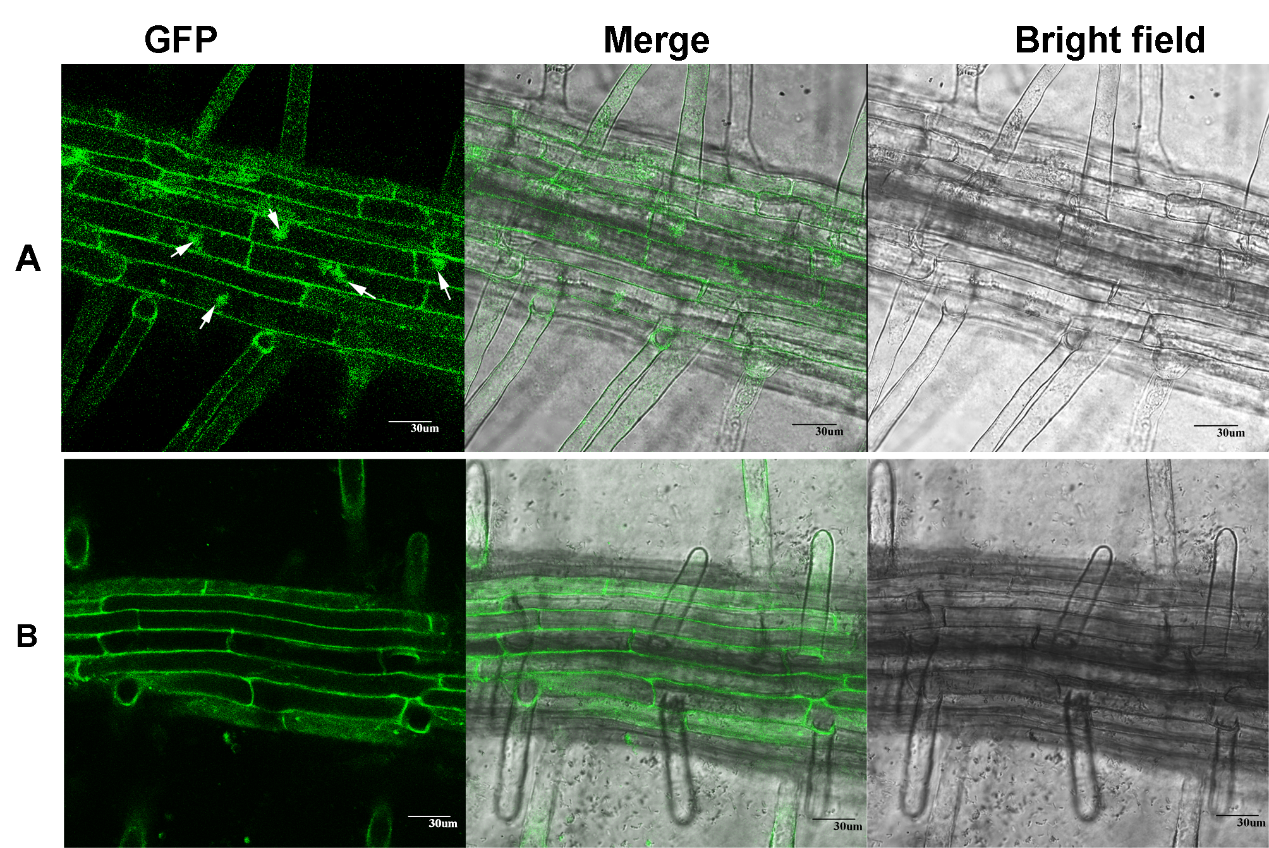


**Supplementary Figure 2. Subcellular localization of GhSNAP33-GFP fusion protein. (A)** Confocal images of eGFP transgenic *Arabidopsis* roots. **(B)** Confocal images of GhSNAP33-GFP transgenic *Arabidopsis* roots. White arrows indicate the GFP fluorescence of nucleus. Scale bar represents 30 μm.

**
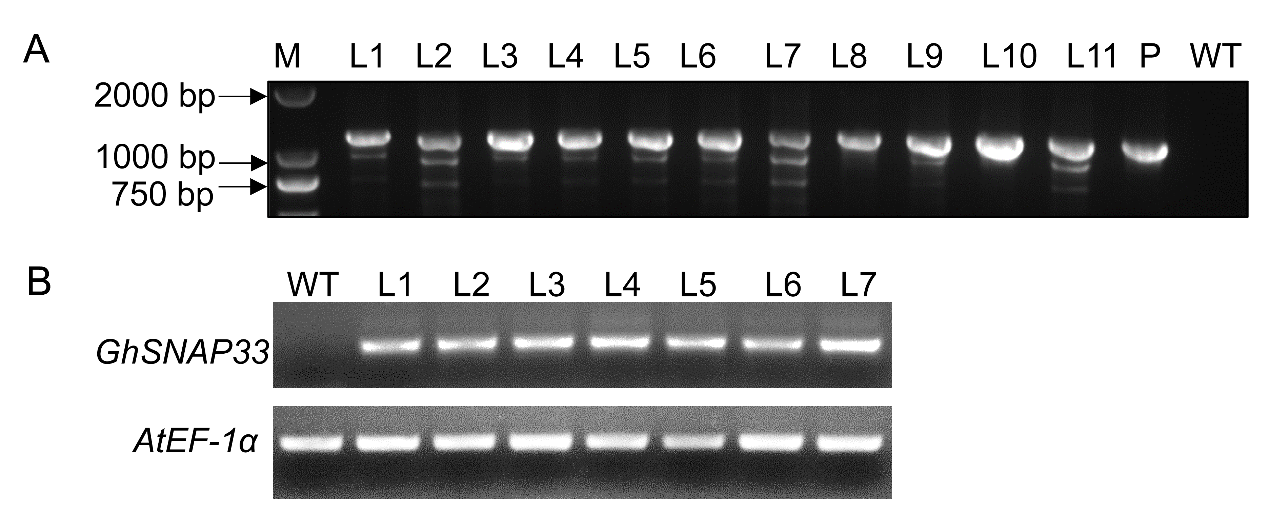
**

**Supplementary Figure 3. Genetic identification of *GhSNAP33* transgenic lines.** (A) Genomic DNA- PCR analysis of genomic DNA from hygromycin B resistant lines; (B) The expression of *GhSNAP33* in transgenic lines by semi RT-PCR.

**Supplementary Table**

| Primer | Sequence (5'→3') |
| --- | --- |
| qUBQ-F | GAAGGCATTCCACCTGACCAAC |
| qUBQ-R | CTTGACCTTCTTCTTCTTGTGCTTG |
| qGhSNAP33-F | GCTGAGGAGACTACAAAGACTG |
| qGhSNAP33-R | ACATACCTCCAAGGCTTCCAAG |
| vGhCLA1-F | CGACGACAAGACCGTGACCATGCACAACATCGATGATTTAG |
| vGhCLA1-R | GAGGAGAAGAGCCGTCATTAGCATGAATGATGAGTAGATTGCAC |
| vGhSNAP33-F | CGACGACAAGACCGTGACCATGACTGTCAATAACTGTGTG |
| vGhSNAP33-R | GAGGAGAAGAGCCGTCATTAGAGTAATCGACGAGCACGTTG |
| ZWSNAP33-F | AGGCTCTAGAATGTCGGGTTTAAAGAGATC |
| ZWSNAP33-R | AAGCGTCGACTCTTTCCGAGTAATCGACGA |
| 1300-F | GCCATTTCGCCTTTTCAG |
| 1300-R | GCTGAACTTGTGGCCGTT |
| pYES-GhSNAP33-F | CCGGAATTCACGATGGCGGGTTTAAAGAG |
| pYES-GhSNAP33-R | ACCGCTCGAGCTACTTTCCGAGTAATCGAC |
| pYES-F | TAATACGACTCACTATAGGG |
| pYES-R | GAATGTAAGCGTGACATAAC |
| qAtEF1α-F | CCTGGATTGCCACACC |
| qAtEF1α-R | AGTCTGCCTCATGTCC |
| qVD-F | AAAGTTTTAATGGTTCGCTAAGA |
| qVD-F | CTTGGTCATTTAGAGGAAGTAA |
| qAtPR1-F | CATACACTCTGGTGGGCCTT |
| qAtPR1-R | CTCACTTTGGCACATCCGAG |
| qAtPR5-F | TCACTCTAGTAGGCGATGGCG |
| qAtPR5-R | GCAGGCCACGACATTGTTCTG |
| qAtRD29A-F | GGAGTACCGGAGATTGCTGA |
| qAtRD29A-R | GCTTCTCGTCGACAAGTCTC |
| qAtDREB2A-F | AAAGGTAAAGGAGGACCAGA |
| qAtDREB2A-R | GCCAAAGGACCATACATAGC |

**Supplementary Table 1.** List of primers used in this study

The enzyme restriction sites were underlined.
